# Editorial: Vesicular biology, the inter- and intra-cellular communicators that shed light on the biology of parasites

**DOI:** 10.3389/fcimb.2023.1141048

**Published:** 2023-02-01

**Authors:** Silvia Castellanos-Castro, Kumiko Nakada-Tsukui, Marcel Ivan Ramirez

**Affiliations:** ^1^ Universidad Autónoma de la Ciudad de México, Colegio de Ciencias y Humanidades, Ciudad de México, Mexico; ^2^ Department of Parasitology, National Institute of Infectious Diseases, Tokyo, Japan; ^3^ Instituto Carlos Chagas Fiocruz Parana, Curitiba, Brazil

**Keywords:** extracellular vesicles, intracellular vesicles, microvesicles, vesicular biology, cell communications, parasite-host interaction, parasites, vesicular traffic

## Introduction

Cellular communications are achieved by releasing and receiving the extracellular vesicles (EVs). Which are bilayered, nanoscale structures that include exosomes (30-100 nm) originated from multivesicular bodies and microvesicles (100-1000 nm) originated from plasmatic membranes. EVs are secreted by almost all cell types into the extracellular environment, released from the cell of origin, to target cells being able to deliver cargo and change the phenotype of the receiving cell (Raposo and Stahl, 2019). Vesicles contain proteins, lipids, nucleic acids, glycoconjugates, and other biomolecules that can be transported intra or extracellularly, defining concepts of vesicles inter-cellular traffic and cellular communication (Yanez-Mo et al, 2015; van Niel et al, 2018).

The vesicle inter-cellular traffic is mediated by the endomembrane system, which include nucleus, endoplasmic reticulum, Golgi apparatus, secretory vesicles, endosomes, and plasma membrane. Furthermore, there is an interplay between vesicles and some varieties of unique organelle which provide an indispensable role in the cell/organism to adapt their environmental niche and fulfill their functions (Bannykh et al., 1996; Barlowe and Miller, 2013).

We propose to understand the biology of vesicular compartmentalized organelle mediated events as “vesicular biology” differentiating between intra and extracellular vesicles. Managing and regulating the vesicle mediated events are centrally important to understand the biology of eukaryotes. In this Research Topic, we would like to take advantage of the vesicular biological point of view to solve the problems in eukaryotic pathogen infection from both parasite and host sides. Currently, advances in molecular biology, biochemistry, -omics approaches and bioinformatics, enables us to find shared molecular events in morphologically different organelles, vesicles and compartments among wide varieties of eukaryotic pathogens including protozoa and helminth. It will help to understand unique and universal vesicular biology and lead to the development of novel therapeutic strategies (Nam, et al, 2020).

In this issue, we have heterogeneous topics including intra and extracellular vesicles and parasitic modes of *Entamoeba histolytica*, *Plasmodium* spp.*, Toxoplasma gondii* and *Fasciola hepatica* showing insights into multiple roles of vesicular biology in diverse cellular functions.

## Membrane trafficking and organelle biogenesis

The inter-cellular traffic of vesicles is known to be mediated by varieties of molecules involved in membrane fission, fusion, and deformation machinery. The major and evolutionarily conserved regulators of these processes are the small GTPases and their accessory molecules (Reiner and Lundquist, 2018). In *E. histolytica*, the member of Arf GTPase family, EhArfX2 plays an important role during the vesicular traffic pathways of virulence factors from trans-Golgi to lysosomes, such as cysteine proteases. It was unexpected but Saito-Nakano et al. identified EhArfX2 as a highly expressed gene in a virulent strain. They revealed a novel role of EhArfx2 that confers to the parasite nitrosative stress resistance during liver abscess development. GTPase proteins from the host cell can be activated by the immune system, and trigger signaling pathways that operate against several pathogenic microorganisms. During *T. gondii* infection, interferon-γ immunity-related GTPases (IRGs) participate in destruction of parasitophorous vacuole restricting intracellular-parasite replication. Using artificial lipid membranes, Yamada et al. demonstrated that Irgb6 directly deformed the membrane and it´s GTPase activity was stimulated by binding to the membrane. Upon GTP hydrolysis, Irgb6 detaches from the membrane, which may promote membrane disruption.

Phagocytosis and trogocytosis are important steps in the mechanism of aggression by *E. histolytica* trophozoites and there are several molecules involved in membrane traffic-related pathways for phagosome biogenesis. Nakada-Tsukui et al., showed that autophagy and phagosomes biogenesis share molecular bases. These authors described the participation of Atg8 during phagocytosis and trogocytosis using comparative proteomic analysis of phagosomes isolated from wild type and *atg8* silenced strains. They showed the localization of identified Atg8 regulated phagosomal proteins recruited to trogosomes providing information to further elucidate molecular events in the Atg8-dependent regulatory network of phagosome/trogosome biogenesis in *E. histolytica*

Endosomal sorting processes are essential for nutrition, capturing and internalization of cargo molecules in mammalian cells. Proteins of the endosomal sorting complexes required for transport (ESCRT) and others have been associated with the molecular basis of the virulence of *E. histolytica* trophozoites. Galindo et al., described the role of Eh-ESCRT-I complex in cell proliferation, phagocytosis, migration and tissue invasion, all of them having an impact during liver abscess formation.These approaches are useful to understand the interconnections of those cellular events that make this parasite an aggressive pathogen.

## Extracellular vesicles and host-parasite interactions

EV studies have been growing to better understand parasite-host communication; it involves the activation of host immune response to eliminate the parasite and on other hand, the evasion of this response by the parasite to survive. Trelis et al., used proteomic approaches to compare the protein content in EV of *F. hepatica* eggs/miracidia and juveniles. They found molecules related to locomotion, membrane traffic and cell physiology, all of them useful to the parasite adaptation to the immune system and host environment during the different development stages. This research provides new data of potential markers to detect the presence of the parasites in their initial developmental stages and for the design of vaccines or antiparasitic drugs

Diaz-Godinez et al., described the aggressive cross talk between *E. histolytica* and neutrophils through comparative proteomic analysis of EVs cargo proteins. Parasitic EVs are able to fuse with neutrophils and affect their functions. In response, neutrophils secrete antimicrobial molecules such as lactotransferrin, myeloperoxidase, elastase and defensins. Thus, both cells attack and manipulate the opposite cell in a distance. Furthermore, NETosis, other attacking processes require contact. These results highlight the importance of EVs in the immunomodulatory effects exerted by amoeba on human neutrophils.

In another parasitic model, *Plasmodium sp*, the malaria-causing agent, the quality of extracellular vesicles of artemyolysin-resistant strains was interestingly studied by Tandoh et al. Through comparative transcriptomes they have seen some molecules that would be involved in the biogenesis of extracellular vesicles of resistant populations, opening possibilities to understand the mechanisms of resistance to the drug.

## Concluding remarks

Knowledge of basic aspects of biogenesis, transport and function on intra and extracellular vesicles can be used to formulate ideas and chemotherapeutic alternatives to control diseases. The plasticity of biological membranes and the enormous role in cellular communication allows ideas for use of them in delivery of vaccines, drugs or conjugation of nucleic acids in therapies.

We have hopes and broad perspectives in the world of vesicles that are in constant revolution and presage advances in basic and applied science.

## Author contributions

All authors listed have made a substantial, direct, and intellectual contribution to the work and approved it for publication.
